# Isolation, Characterization, and Wound‐Healing Potential of *β‐D*‐Glucan from *Lycoperdon pyriforme* Schaeff

**DOI:** 10.1002/open.202500131

**Published:** 2025-07-27

**Authors:** Elif Yavuz‐Dokgöz, Meltem Güleç, Burçin İzbudak, Onur Şahin, Ayça Bal‐Öztürk

**Affiliations:** ^1^ Department of Biochemistry Faculty of Pharmacy Istinye University Sariyer 34396 Istanbul Türkiye; ^2^ Department of Pharmacognosy Faculty of Pharmacy Istanbul University‐Cerrahpaşa Buyukcekmece 34500 Istanbul Türkiye; ^3^ Department of Pharmacognosy Faculty of Pharmacy Istinye University Sariyer 34396 Istanbul Türkiye; ^4^ Department of Stem Cell and Tissue Engineering Institute of Graduate Education Istinye University Sariyer 34396 Istanbul Türkiye; ^5^ Department of Pharmaceutical Chemistry Faculty of Pharmacy Istinye University Sariyer 34396 Istanbul Türkiye; ^6^ Department of Analytical Chemistry Faculty of Pharmacy Istinye University Sariyer 34396 Istanbul Türkiye; ^7^ Stem Cell and Tissue Engineering R&D Center (ISUKOK) Istinye University Sariyer 34396 Istanbul Türkiye

**Keywords:** hemocompatibility, *Lycoperdon pyriforme*, wound healing, *β*‐Glucan

## Abstract

Mushrooms have become a prominent focus in alternative medicine and are now the subject of increasingly detailed scientific investigation. This study explores the isolation, purification, and characterization of *β*‐*D*‐glucan from *Lycoperdon pyriforme*, with particular emphasis on its wound‐healing potential. The aim of this research is to isolate, purify, and characterize *β‐*glucan compounds from mushrooms collected in their natural habitat. Gel permeation chromatography (GPC), Fourier transform infrared spectroscopy (FTIR), and nuclear magnetic resonance (NMR) spectroscopy are employed to characterize the compound. In vitro cytotoxicity is assessed using NIH 3T3 cells, and surface morphology is evaluated via electron microscopy. Spectroscopic analyses (GPC, FTIR, and NMR) confirmed the presence of *β*‐(1 → 3) and *β*‐(1 → 6) linkages and indicated polydispersity in the isolated *β‐D‐*glucan. Cytotoxicity assays show no toxic effect on NIH 3T3 cells, even at 1000 μg/mL. Lower concentrations (20, 100, and 500 μg/mL) promoted cell migration and wound closure in scratch assays. Hemolytic activity remained below 5%. The findings suggest that *β‐D*‐glucan from *Lycoperdon pyriforme* may support wound healing due to its non‐toxic, hemocompatible, and coagulation‐promoting properties. Future studies will focus on elucidating its mechanisms of action and evaluating clinical efficacy.

## Graphical abstract

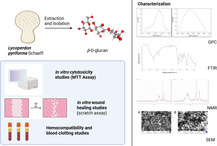

## 1 Introduction

Mushrooms, belonging to the kingdom Fungi, encompass a diverse array of species with varied ecological roles and applications. Bioactive substances used in complementary and alternative medicine naturally occur in mushrooms, making them a valuable resource. Since ancient times, people have consumed wild mushrooms as a delicacy, likely due to their taste and appealing flavor.^[^ ^1^ ^]^ Today, the prevalence of alternative medicine is increasing in parallel with the number of diseases. In this context, mushrooms, which alternative medicine is oriented toward, have recently been investigated in more detail.^[^ ^2^, ^3^ ^]^

*Lycoperdon,* which evidently exhibits a gasteromycete‐like habit, was already recognized during the pre‐Linnaean era as a species due to its frequent presence and significant size, typically measuring around 10 mm in diameter.^[^ ^4^ ^]^ The saprobic fungus *Lycoperdon pyriforme* Schaeff., which is also known as the pear‐shaped puffball or trunk puffball, is found worldwide, commonly on slumps and roots, sometimes even on soil, on dead, generally rotting wood of broadleaved trees and conifers, always connected to wood by mycelial threads.^[^ ^5^ ^]^ Mushrooms are white when young, turn yellowish‐brown as they mature, and can range from 2 to 6 cm in diameter. Reproduction takes place via spores that spread through the opening in the underside of the mature mushroom. Because the spores of *L. pyriforme* are so dry, when mature, they resemble a fine sack of dust. Due to its anti‐coagulation qualities, this powder has been utilized in several ancient societies to aid in the healing of wounds. Especially the spores were previously utilized in medicinal practices by native people in India and northern Europe.^[^ ^6^ ^]^ *Lycoperdon pyriforme* is also documented as being used in traditional medicine by indigenous populations in several regions of Ethiopia.^[^ ^7^ ^]^ However, because various agaricomycetes have somewhat varied properties, it is crucial to choose a real *L. pyriforme*. As an illustration, *Filobasidiella neoformans* is dangerous for people with impaired immune systems.^[^ ^8^, ^9^ ^]^

Common polysaccharides found in cereals and microbes are called *β*‐glucans. The Food and Drug Administration (FDA) authorized the usage in the pharmaceutical business, as well as for safe application as a food ingredient and nutraceutical.^[^ ^10^ ^]^ *β*‐Glucans exhibit significant biological activity, particularly in the area of immunoregulation. Over time, there has been an increasing focus on the effects of *β*‐glucan. Today, it has been proven that *β*‐glucans, which are connected by *β*‐(1,3)‐glycosidic bonds and contain varying proportions of *β*‐(1,4) and *β*‐(1,6)‐glycosidic side chains, exhibit exceptional immunoregulatory, hypoglycemic, anticancer, cholesterol‐lowering, and anti‐infectious properties. Numerous earlier studies regarding *β*‐glucans have shown encouraging properties. The body uses α‐ and *β*‐glucans, which are indigestible fibers with clear physiological activities, as its energy source. They are mostly found as α‐ and *β*‐(1 → 4)‐linked linear structures between glucose residues (cellulose and amylose, respectively). Based on its three‐dimensional conformation, *β*‐glucan, also known as (1 → 3)‐*β*‐glucan, can be classified as a single, triple, or random helix (irregular helix).^[^ ^10^, ^11^ ^]^ Biological functions of fungal *β*‐glucans are related to wound‐healing activities. Fungal *β*‐glucans have been found to exhibit various biological functions such as immunomodulatory, anti‐inflammatory, antioxidant, antiviral, antitumor, antiobesity, and antidiabetic.^[^ ^12^ ^]^ Fungal *β*‐glucans are important molecules for wound healing in general. Although fractional lasers have been shown to be an important tool for skin repair, they can lead to excessive inflammatory response and skin barrier dysfunction.^[^ ^13^ ^]^ Therefore, it has been reported that an effective strategy should be developed to promote wound healing after fractional laser treatment.^[^ ^14^ ^]^ Cao et al. examined the wound‐healing effect of *β*‐glucan from *Schizophyllum commune* on patients receiving fractional laser therapy. The results showed that *β*‐glucan in *Schizophyllum commune* significantly reduced the inflammatory response and improved skin erythema. Thus, it accelerated the *wound‐healing* process of patients after fractional laser treatment. The potential mechanism has been reported as the ability of *β*‐glucan to bind with the Dectin‐1 receptor and subsequently stimulate the expression of various immune system‐related cytokines.^[^ ^15^ ^]^ Numerous investigations have demonstrated that fungal *β*‐glucans can stimulate cell migration and proliferation in vitro. In their 2018 study, de Jesus et al. examined the effectiveness of a *β*‐glucan derived from *Piptoporus betulinus* mushrooms in healing wounds.^[^ ^8^ ^]^ Sponges show great potential as wound‐healing materials. The high porous structure of sponges is convenient for gas and nutrient permeation and can provide a matrix for cell proliferation.^[^ ^16^ ^]^ More importantly, sponges can quickly absorb water in blood, thereby leading to accumulation of blood coagulation factors and quick hemostasis. Therefore, sponges are suitable for acute or highly exudative wounds. Sun et al. reported a chitin‐glucan sponge obtained from *Pleurotus eryngii*. The *Pleurotus eryngii* fruit bodies were treated with alkali to remove the water‐soluble protein and glucan.^[^ ^17^ ^]^

In recent studies, *β*‐glucans from different fungal species have been obtained and used in applications for the treatment of different types of wounds. Fungal *β*‐glucans may promote fibroblast proliferation, angiogenesis, collagen deposition, and re‐epithelialization in the wound‐healing process. Some studies even suggest that mushroom *β*‐glucans may promote the polarization of macrophages from the M1 to the M2 phenotype. All these studies indicate that *β*‐glucans isolated from fungi have a good wound‐healing ability.^[^ ^16^ ^]^

The *Lycoperdon pyriforme* sample we used in our study has long been utilized by local communities on the Sultan Murat Plateau (Çaykara, Trabzon) to promote wound healing and reduce bleeding. Traditionally, the dried fruiting body of the mushroom is directly applied to skin injuries. Despite its continued use in folk medicine, no prior scientific documentation of this ethnomedicinal practice exists in the literature. Therefore, the present study aims not only to investigate the therapeutic properties of *β‐D‐*glucan derived from *L. pyriforme* but also to provide the first evidence‐based record of its traditional use. By doing so, this work bridges a significant gap between local ethnobotanical knowledge and modern biomedical research. To the best of our knowledge, this is the first study to provide both biochemical characterization (GPC, Fourier transform infrared spectroscopy (FTIR), and NMR) and functional biological evaluation (wound healing, cytotoxicity, hemocompatibility, and coagulation) of *β‐D*‐glucan isolated from *Lycoperdon pyriforme*.

## 2 Experimental Section

### 2.1 Materials

**Figure 1:**
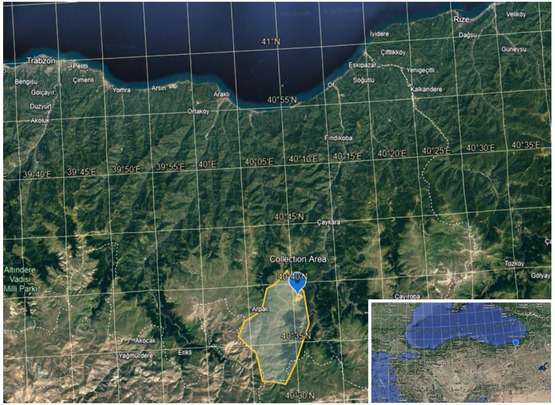
Geographical region where mushroom samples were collected. Image © 2025 Google, Image Landsat/Copernicus.

*Lycoperdon pyriforme* was collected from Sultan Murat Plateau (Çaykara, Trabzon, Türkiye) (**Figure** **1**). The collected specimen was identified by Dr. Selin Aktar Kiremitci based on its characteristic morphological features.^[^ ^18^, ^19^ ^]^ The geographical area where the mushrooms are collected can be up to 2500 meters above sea level (Supporting Information 1). The samples collected from the field were air‐dried at room temperature in a well‐ventilated environment, protected from direct sunlight. All chemicals needed for the study were purchased from Sigma (St. Louis, MO, USA).

Dulbecco's Modified Eagle Medium High Glucose (DMEM High Glucose) and fetal bovine serum (FBS) were purchased from Capricorn. DPBS from PAN, *L*‐glutamine, penicillin/streptomycin and 0.05% trypsin/0.02 EDTA were purchased from Gibco, respectively. 3‐(4,5‐dimethylthiazol‐2yl)‐2,5‐diphenyltetrazoliumbromide (MTT) was purchased from Biomatik.

### 2.2 Extraction and Purification

The extraction and purification procedures were performed with minor modifications based on the method described by de Jesus et al. (2018). To eliminate nonpolar substances, the dried and ground *Lycoperdon pyriforme* sample (57 g) was extracted with chloroform‐methanol (2:1 v/v) at 60 °C for 3 h, repeated 3 times. The resulting organic extract was then concentrated under reduced pressure using a rotary evaporator at ≈70 °C and 150 mbar to efficiently remove residual solvents. The remaining pulp was subsequently air‐dried overnight at room temperature (≈25 °C) in a well‐ventilated environment to ensure complete removal of moisture prior to aqueous extraction.

**Figure 2:**
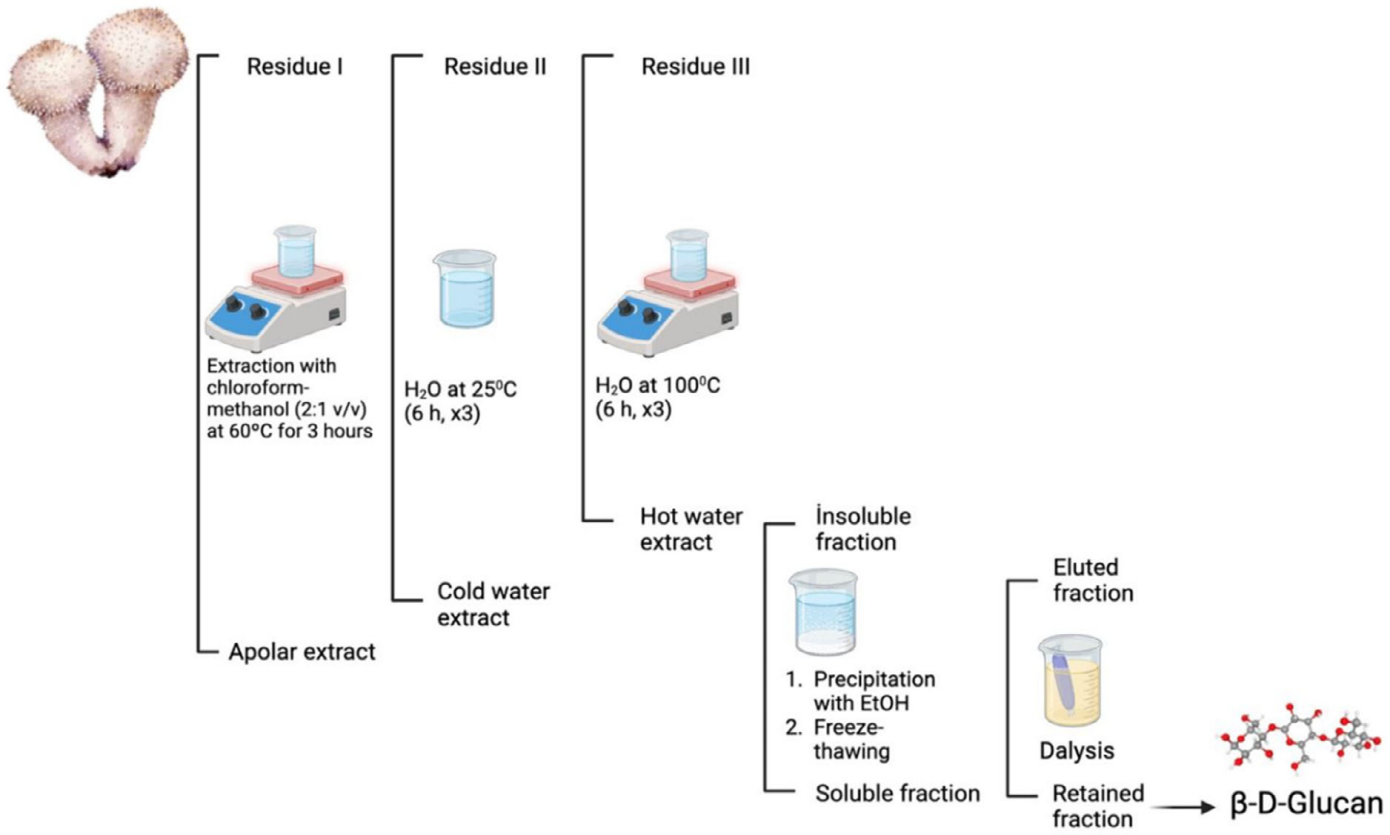
Procedure of isolating *β*‐*D*‐glucan.

Sequential aqueous extractions were then performed. First, the sample was extracted with cold water at 25 °C for 6 h, repeated three times. The residual pulp was subsequently extracted with hot water at 100 °C, also for 6 h, and repeated three times. The combined water‐soluble fractions were concentrated under reduced pressure using a rotary evaporator. The polysaccharide was then collected by the addition of ethanol (3:1 v/v). After centrifugation at 8000 rpm at 10 °C for 20 min, the sediment was dialyzed against distilled water (pH ≈ 7.0) for 24 h using a 6–8 kDa Mr cut‐off membrane. The final material was concentrated under decreased pressure and lyophilized for storage (**Figure** **2**).

### 2.3 Characterization of Isolated *β‐D‐Glucan*

The molar mass distribution of the isolated *β‐D‐glucan* from the mushroom was analyzed by GPC, Waters Ultrahydrogel 250 and 500. The flow rate is 0.7 mL min^−1^. The concentration is 5 mg mL^−1^ in phosphate buffer (pH 7.2). The FTIR spectra of the extracted *β*‐glucan were recorded on a Thermo Nicolet 6700 FTIR spectrometer at room temperature in the wavelength region between 4000 and 400 cm^−1^. The NMR spectrum was recorded on a 500 MHz Agilent VNMRS. Analysis was performed in D_2_O. Chemical shifts are expressed in ppm. Morphological characteristics of isolated *β*‐*D*‐glucan were investigated by SEM analysis. Samples were coated with gold, and SEM images were taken using scanning electron microscopy (Zeiss EVO LS 10), operated at 20 kV.

### 2.4 Cell Culture

NIH 3T3 (ATCC CRL‐1658) cells were maintained in DMEM high glucose supplemented with 10% v/v FBS, 1% v/v L‐glutamine, and 1% v/v penicillin‐streptomycin (10 000 U mL^−1^) under standard cell culture conditions. The cultures were maintained at 37 °C in a humidified atmosphere with 5% CO_2_. The culture medium in 75 cm^2^ cell culture flasks was refreshed every three days. Upon reaching 90% confluency, NIH 3T3 cells were detached using a trypsin‐EDTA (0.05%), phenol red solution and resuspended in DMEM high glucose. The cell suspension was centrifuged at room temperature for 5 min at 300 g. Following the removal of the supernatant, the cells were resuspended in DMEM high glucose and subsequently counted using a Thoma hemocytometer. Passage 12 NIH 3T3 cells were utilized in the experiments.

### 2.5 MTT Assay

An indirect MTT assay was employed to evaluate the cytotoxic effects of varying concentrations of mushroom extract (20 μg, 100 μg, 500 μg, and 1000 μg) on cells. Samples of these concentrations were weighed, prepared in DMEM high glucose medium, and sterilized using a 0.45 μm syringe filter. NIH 3T3 cells were seeded at a density of 7 × 10^3^ cells per well in a 96‐well plate, with each well containing 100 μL of DMEM high glucose, and were initially incubated for 24 h at 37 °C in a humidified atmosphere with 5% CO_2_ to allow cell attachment and stabilization. Following this incubation period, the culture medium was carefully replaced with varying concentrations of the mushroom extract, and the cells were further incubated under the same conditions for an additional 24 h to assess the effects of the extract on cell viability and behavior. Following this, 100 μL of DMEM high‐glucose and 10 μL of MTT solution (5 mg mL^−1^ in PBS) were added to each well and incubated at 37 °C for 4 h under 5% CO_2_ to allow formazan crystal formation.^[^ ^20^ ^]^ Following the incubation period, the DMEM high‐glucose medium containing 10% MTT was carefully removed, and 100 μL of DMSO was added to each well to facilitate the dissolution of formazan crystals, ensuring optimal solubilization. The plates were then incubated at 37 °C for 20 min to allow complete dissolution, after which the optical density was measured at 570 nm using a microplate reader (BMG Spectrostar) to quantitatively assess cell viability. As a control group, cells were cultured in DMEM high‐glucose medium without any sample treatment.

### 2.6 Scratch Assay

The in vitro scratch assay was performed to investigate the potential effects of mushroom extracts on wound healing. NIH 3T3 fibroblast cells were initially seeded at a density of 2.5 × 10^5^ cells per well in a 24‐well plate and allowed to be cultured until a uniform, confluent monolayer was established. A vertical scratch was carefully introduced using a 200 μL pipette tip to simulate a wound. Detached cells and residual debris were then thoroughly removed by washing with phosphate‐buffered saline (PBS), ensuring a clean wound area. Subsequently, the prepared mushroom extracts were applied to the wells following the methodology outlined for the MTT assay, ensuring consistency in experimental conditions. The treatment medium consisted of DMEM high glucose supplemented with 10% v/v FBS, 1% v/v L‐glutamine, and 1% v/v penicillin‐streptomycin. Wells containing only medium served as untreated controls, while others were treated with varying concentrations of mushroom extracts. All plates were then incubated at 37 °C in a humidified atmosphere with 5% CO_2_ to facilitate cellular responses to the applied extracts. To monitor the progression of wound closure over time, images were systematically captured at predefined intervals (0, 5, 10, 24, and 48 h) using an inverted microscope (Zeiss AxioScope Z1) and synchronized for further analysis. The extent of wound closure was quantified using ImageJ (NIH) software, and the percentage of wound closure was subsequently calculated based on Equation (1).

(1) $$\text{Wound Closure }\left(\%\right)=\frac{T_{0}-T_{X}}{T_{0}}\times 100$$

In this equation, *T*_0_ corresponds to the initial wound area measured immediately after the scratch was created, while *T*_x_ represents the wound area at a given time point during the observation period.

### 2.7 Hemolytic Activity Assay

The hemocompatibility of mushroom extracts at varying concentrations was systematically evaluated by assessing their interaction with erythrocytes. The bovine blood obtained from the slaughterhouse was first centrifuged at 3000 rpm for 10 min to separate the serum and isolate the erythrocytes. Subsequently, the erythrocytes were washed three times with sterile PBS buffer to remove any remaining plasma components and then diluted to a final concentration of 5% v/v to standardize the experimental conditions.^[^ ^21^ ^]^ Meanwhile, mushroom extracts were prepared at different concentrations in PBS (20 μg mL^−1^, 100 μg mL^−1^, 500 μg mL^−1^, and 1000 μg mL^−1^) to investigate the dose‐dependent effects on erythrocyte integrity. Following preparation, 500 μL of the erythrocyte suspension was gently mixed with 500 μL of each sample dispersion in 2 mL tubes to ensure uniform interaction, after which the mixtures were incubated at 37 °C for 90 min to allow potential hemolytic activity to occur. Upon completion of the incubation period, the samples were centrifuged at 3500 rpm for 10 min to pellet intact erythrocytes, and 100 μL of the resulting supernatant was carefully transferred to a 96‐well culture plate for further analysis. The hemoglobin content in the supernatant, indicative of hemolysis, was quantitatively measured using a microplate spectrophotometer set at 540 nm, enabling an accurate assessment of erythrocyte membrane disruption. To establish baseline comparisons, distilled water and PBS were used as the positive and negative controls, respectively, while the percentage of hemolysis for each sample was determined using Equation (2).

(2) $$\text{Hemolysis }\left(\%\right)\;=\;\left(A_{\text{sample}}-A_{\text{negative control}}\right)/(A_{\text{positive control}}-A_{\text{negative control}})\times 100\%$$

### 2.8 Whole‐Blood Clotting Assay

To evaluate the effects of mushroom extracts on blood coagulation, bovine whole blood was obtained from a slaughterhouse. To prevent premature clotting and to maintain blood stability throughout the assay, 10 mL of whole blood was mixed with 1 mL of sodium citrate solution (38 mg mL^−1^) in a centrifuge tube. In parallel, 80 μL of mushroom extracts at varying concentrations was carefully combined with 80 μL of the citrated blood sample in an Eppendorf tube to ensure uniform mixing, after which the mixture was maintained at 37 °C to replicate physiological conditions. To initiate the coagulation process, 6 μL of CaCl_2_ solution (0.2 m) was added, thereby counteracting the anticoagulant effect of sodium citrate and triggering clot formation. Following the designated incubation period, during which coagulation was allowed to proceed, 1 mL of water was gently introduced to dissolve any unbound blood components and stabilize the clot structure, preventing its disintegration during subsequent handling. After ensuring complete stabilization, 100 μL of the supernatant was carefully transferred to a 96‐well culture plate to facilitate quantitative analysis, and its absorbance at 540 nm was measured using a microplate spectrophotometer, enabling precise assessment of clot formation and hemoglobin release. To establish a reference for comparison, the absorbance of recalcified citrated whole blood (80 μL) in 1 mL of water was used as a control, while the blood coagulation index (BCI) of the samples was calculated based on Equation (3).

(3) BCI (%)=A/B×100%

where *A* represents test group absorbances and *B* represents reference value absorbances (negative control group).

## 3 Results and Discussion

### 3.1 Results

#### 3.1.1 Lycoperdon pyriforme Chemical Analysis

The chemical characterization of the mushroom was supported by three spectroscopic methods; GPC, FTIR, and ^1^H NMR.

#### 3.1.2 Gel Permeation Chromatography (GPC)

**Figure 3:**
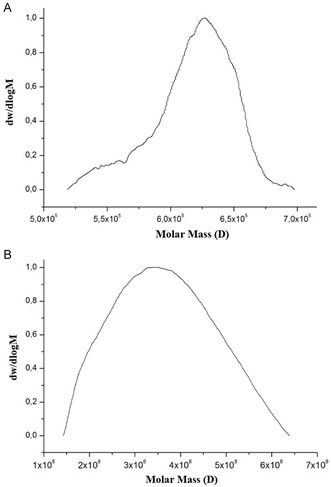
Images of molecular weight distribution in GPC. A) shows GPC result between 13 and 17 min fraction and B) shows GPC result between 20 and 26 min fraction.

The molar mass distribution of the isolated *β*‐*D*‐glucan from the mushroom was analyzed by GPC, Waters Ultrahydrogel 250 and 500. The flow rate is 0.7 mL min^−1^. The concentration is 5 mg mL^−1^ in phosphate buffer (pH = 7.2). As depicted in the GPC chromatogram, two peaks were observed with the number average molecular weights *M*_n_ = 3 kDa and *M*_n_ = 104.55 kDa and polydispersity indices *M*_w_/*M*_n_ = 1.11 and Mw/*M*_n_ = 1.78, respectively. The images of the distribution are as given below (**Figure** **3**). This molecular weight distribution was found to be consistent with the literature.^[^ ^22^ ^]^

#### 3.1.3 FTIR Spectroscopy

**Figure 4:**
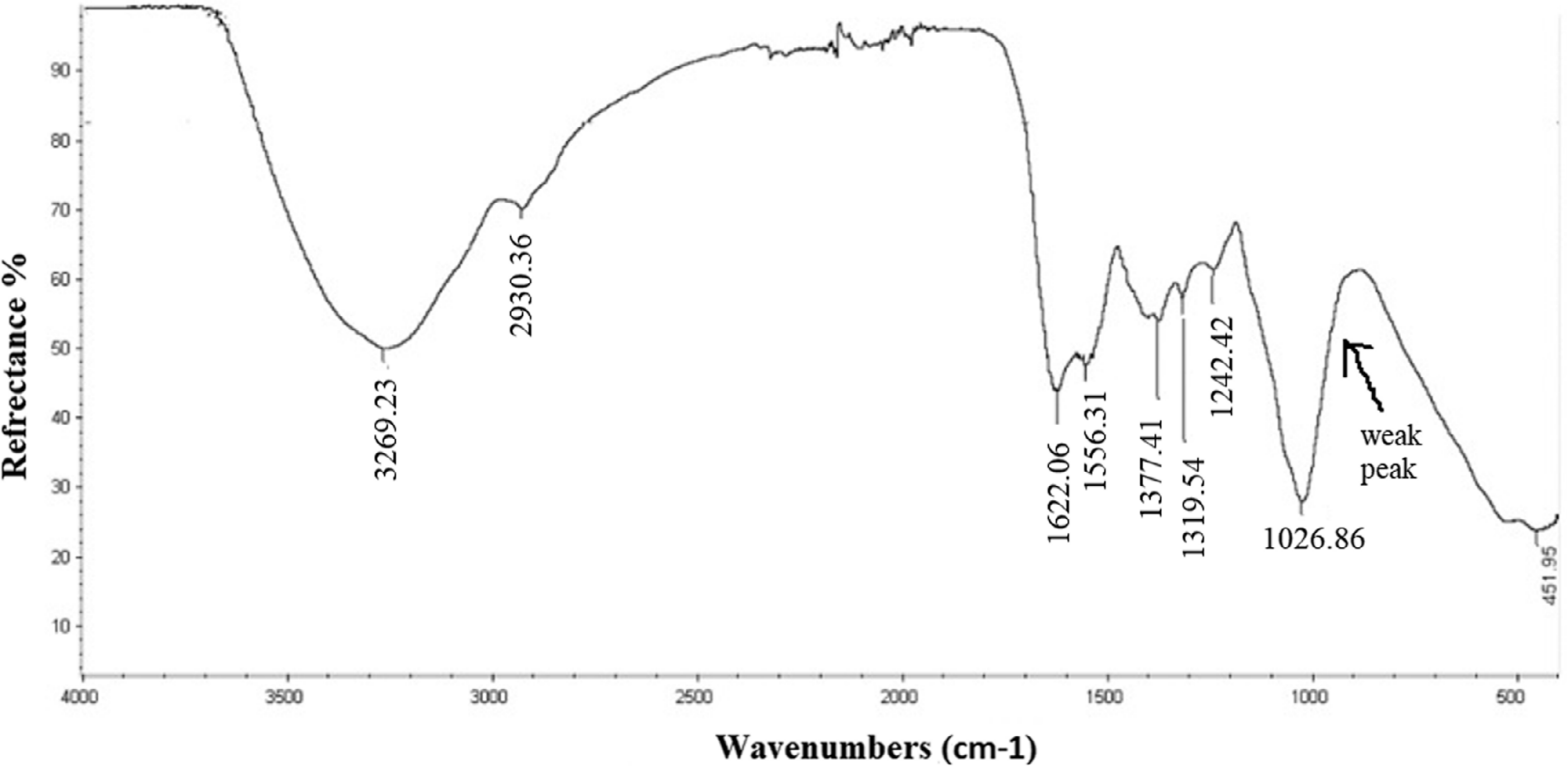
The FTIR spectrum of *β*‐glucan extracted from *Lycoperdon pyriforme.*

The FTIR spectra of the extracted *β*‐glucan were recorded on a Thermo Nicolet 6700 FTIR spectrometer at room temperature, within the range of 4000–400 cm^−1^. Characteristic absorption peaks were observed (**Figure** **4**) at 3269.2, 2930.3, 1622, 1556.3, 1377.4, 1319.5, 1242.4, and 1026.8 cm^−1^. The spectral regions between 1200–950 cm^−1^ (sugar region) and 950–750 cm^−1^ (anomeric region) are particularly significant for the structural analysis of polysaccharides.^[^ ^23^ ^]^

The broad absorption band at 3269.2 cm^−1^ corresponds to O–H stretching vibrations, indicating the presence of hydroxyl groups. The weaker band at 2930.3 cm^−1^ is attributed to C–H stretching vibrations.^[^ ^24^ ^]^

Notably, the absorption peaks at 1377.4, 1319.5, and 1026.8 cm^−1^, along with a subtle shoulder near 900 cm^−1^, are consistent with the presence of *β*‐glucan in the sample. This shoulder, although not sharp, is detectable and is attributed to the C–H deformation of (1 → 3)‐*β‐D*‐glucan linkages, as supported by previous reports.^[^ ^23^, ^25^ ^]^ The bands at 1622 and 1556.3 cm^−1^ correspond to amide I and amide II vibrations, respectively, suggesting the presence of amide functional groups in the extract. Altogether, the FTIR signals strongly support that the isolated material predominantly consists of *β‐D*‐glucan.

#### 3.1.4 Nuclear Magnetic Resonance (NMR) Spectroscopy Studies

The ^1^H NMR spectrum was recorded on a 500 MHz Agilent VNMRS instrument using D_2_O as the solvent. Chemical shifts are expressed in δ (ppm). A water‐soluble *β*‐*D*‐glucan was obtained from *Lycoperdon pyriforme* by the extraction. Unlike cereal‐derived *β*‐glucans, fungal *β‐*glucans typically possess linear *β*‐(1 → 3) glycosidic linkages with occasional *β*‐(1 → 6) branching but lack *β*‐(1 → 4) linkages.^[^ ^26^ ^]^ For the structural characterization of *β*‐glucans, several analytical methods have been reported, including LC‐MS, ^1^H NMR, FTIR, SEM, TEM, DLS, and UV‐visible spectroscopy. One recent study successfully characterized *β*‐glucan without the support of ^13^C‐NMR.^[^ ^27^ ^]^ In that report, typical ^1^H NMR signals were observed at 2.5, 2.7, 3.5, 4.7, 5.2, and 5.3 ppm, confirming the aliphatic glucose structure of *β*‐glucans.

In our spectrum, similar chemical shifts were observed at 2.53, 2.78, 3.53, 4.70, 5.22, and 5.66 ppm. The region between 2 and 5 ppm corresponds to aliphatic –OH proton signals. Signals between 3.3 and 3.8 ppm can be attributed to methoxy protons (R–O–CH_3_), while the anomeric protons appeared at 4.59 and 4.32 ppm, indicating the presence of *β*‐(1 → 3) and *β*‐(1 → 6) linkages, respectively. These findings are consistent with the literature.^[^ ^28^ ^]^

**Figure 5:**
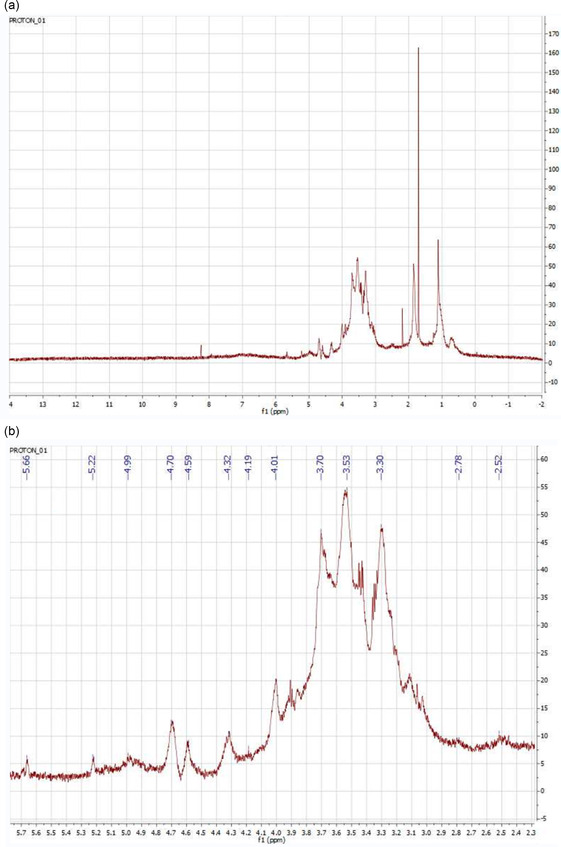
^1^H NMR spectrum of *β*‐glucan extract. a) Full ^1^H‐NMR spectrum of *β‐D*‐glucan isolated from *Lycoperdon pyriforme* and b) magnified ^1^H‐NMR spectrum (between 2.30 and 5.70 ppm) of *β‐D*‐glucan with annotated peaks.

Additionally, a characteristic signal for *β*‐(1 → 3)‐*D*‐glucan was previously reported at 4.55 ppm,^[^ ^29^ ^]^ which aligns well with our results. Other typical anomeric proton signals observed in our ^1^H NMR spectrum include peaks at 4.70, 4.59, 4.19, and 4.01 ppm, consistent with previously reported data.^[^ ^30^ ^]^ (**Figure** **5**).

#### 3.1.5 SEM Analysis

**Figure 6:**
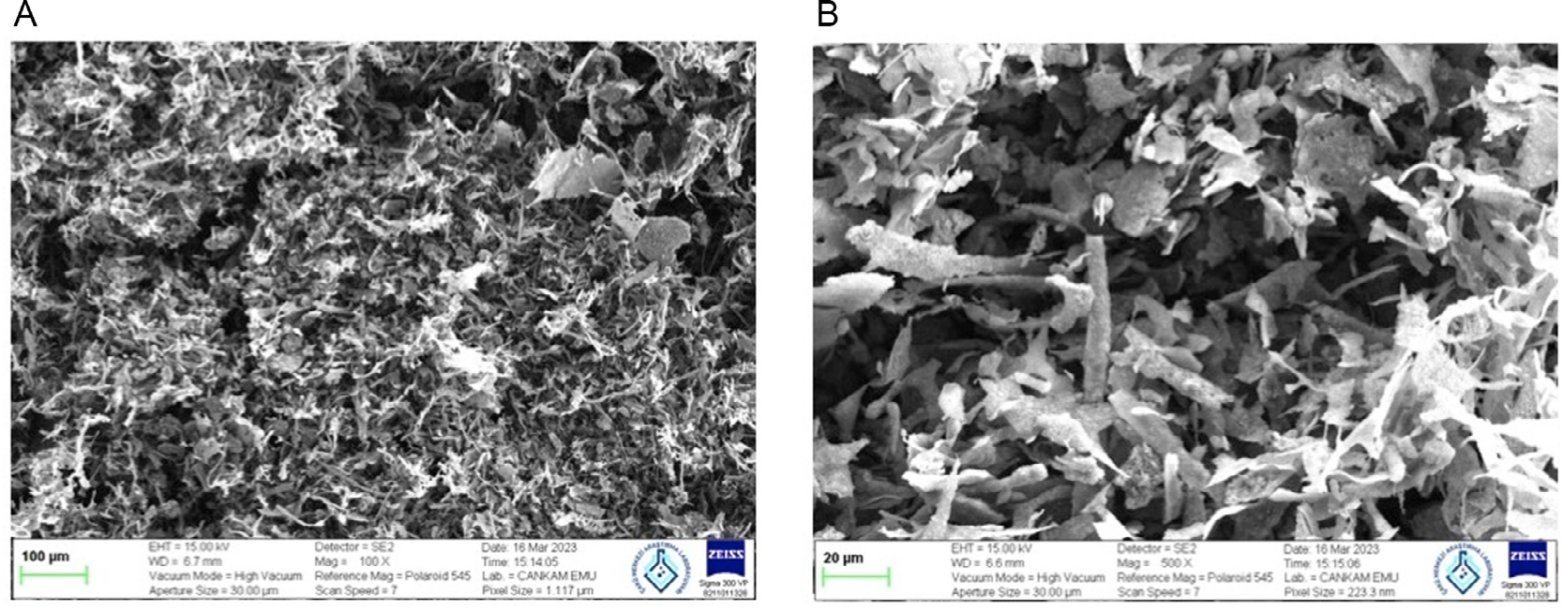
SEM images of the *β*‐glucan extract at different magnifications: A) 100 × magnification with a scale bar of 100 μm, and B) 500 × magnification with a scale bar of 20 μm.

**Figure** **6**A is captured by a 100 × magnification. The image illustrates a layout that is both intricate and compact, spanning a substantial area with the surface morphology, which includes fibrous structures and particles. The presence of these characteristics suggests a potentially porous nature and a high surface area. Figure 6B is captured at 500× magnification for reference and provides a more comprehensive surface analysis due to its increased magnification. The structures appear more detailed and prominent. In this image, fibrous and particle features reveal surface morphology. Larger, more visible particles demonstrate the diversity of the material. In the presence of irregular structures, the material may exhibit insufficient bonding and be brittle. Rough, fibrous, and particulate surfaces are present in both photographs (Figure 6). This implies a sizeable surface area, which may be advantageous for applications that require high adsorption values.

According to the literature, drying technique significantly affects the physiochemical behaviors of *β*‐glucan samples.^[^ ^31^ ^]^ In addition, the different surface topography of *β*‐glucans isolated from the mushroom is due to the difference in the nature of the fruiting bodies among wild and cultivated varieties of mushrooms.^[^ ^32^ ^]^ In general, the particles of standard *β*‐glucan are uniform, irregular aggregations forming irregular masses with spherical to rounded particles.^[^ ^33^ ^]^ Glucan extracted from black fungus has parallel fibril morphology.^[^ ^34^ ^]^ The particles of *β*‐glucan extracted from *Saccharomyces cerevisiae* are irregular (geometric shape) with sharp edges, and aggregated particles form a large mass.^[^ ^33^ ^]^ In this study, *β*‐glucan was extracted from *Lycoperdon pyriforme* and subsequently dried using a lyophilization process. SEM micrographs revealed that the extracted *β*‐glucan exhibited a rough, fibrous, and particulate surface morphology. The noticeable agglomeration of fibrous structures observed in the images is likely attributed to the natural tendency of *β*‐glucan chains to form intermolecular hydrogen bonds during freeze‐drying, which promotes clustering and aggregation.^[^ ^35^ ^]^ This observation is consistent with the literature, which indicates that such structural behavior commonly occurs in natural polysaccharides during drying processes.^[^ ^36^ ^]^

#### 3.1.6 In Vitro Cytotoxicity Studies

**Figure 7:**
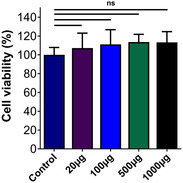
The cell viability of NIH 3T3 treated with mushroom extracts and cells cultured in DMEM high‐glucose medium as control. Results are presented as means ± SD, *n* = 7 (ns: *p* > 0.05).

The absence of cytotoxic effects of mushroom extracts on NIH 3T3 cells was examined by performing the indirect MTT test. Mushroom extracts at different concentrations showed over 100% cell viability in NIH 3T3 cells. It was observed that increasing mushroom extract concentrations did not cause a negative increase in cell viability or proliferation rate (**Figure** **7**). The increase in the concentration of mushroom extracts did not show a specific cell viability effect on cells. There was no cytotoxic effect on cells from 1000 μg mL^−1^ mushroom extract, and cell viability was preserved.

#### 3.1.7 In Vitro Wound‐Healing Studies

**Figure 8:**
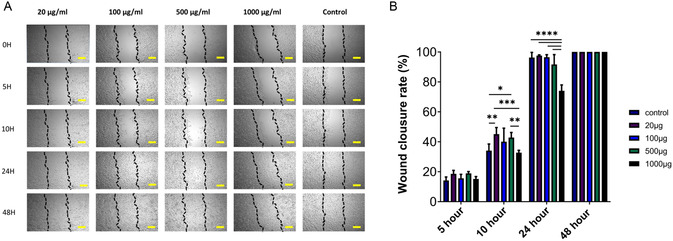
A) The effect of *β*‐*D*‐glucan in the content of mushroom extract wound healing induction. Cell migration after exposure to the extracts (5 × magnification, scale bar: 200 μm). B) Time graph of change of wound area treated with mushroom extract. According to the results of the two‐way ANOVA test, values are presented as mean ± standard deviation (SD). Statistical significance is indicated as follows: no line (*p* > 0.05, not significant), *p ≤ 0.05, **p ≤ 0.01, ***p ≤ 0.001, and ****p ≤ 0.0001.

The effect of *β*‐*D*‐glucan in the content of mushroom extract on wound healing was investigated on NIH 3T3 cells by the in vitro scratch assay. The effect of lyophilized mushroom extract on wound healing at increasing concentrations was examined, and it was observed that the wound pattern was closed at 48 h in all groups (**Figure** **8**A). A faster wound healing was observed in the first 10 h in the groups with 20 μg mL^−1^, 100 μg mL^−1^, and 500 μg mL^−1^ mushrooms compared to the control group in Figure 8B. The *β*‐*D*‐glucan in the mushroom content provided closure of 40%–50% of the wound model tissue in 10 h. While the scar tissue containing 1000 μg mL^−1^ mushroom concentration showed a slower closure pattern than the control group, all of the scar tissue was closed at the end of 48 h. It has been defined that *β*‐*D*‐glucans have an effect on monolayer cell regeneration and increase cell migration and proliferation.^[^ ^8^ ^]^ Similarly, Woo et al. have shown that *β*‐(1 → 3), (1 → 6)‐D‐glucan can directly increase fibroblast migration and proliferation on human dermal fibroblast cells, potentially offering significant effects on the wound‐healing process. The fact that it is effective even in the absence of serum suggests that *β*‐glucan may directly interact with extracellular signaling.^[^ ^37^ ^]^

#### 3.1.8 Hemocompatibility and Blood Clotting Studies

**Figure 9:**
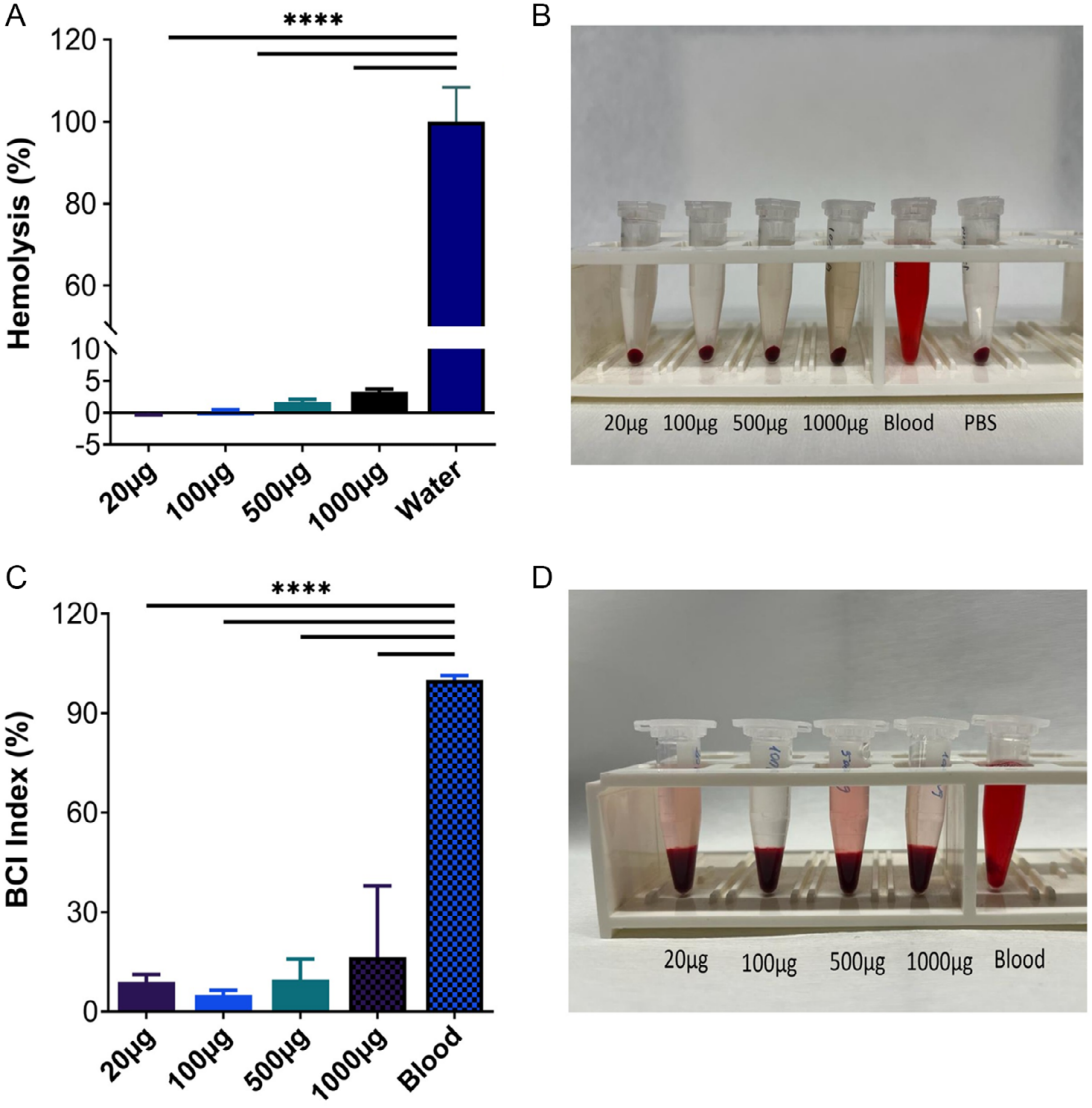
A) Hemolytic activity results of mushroom extracts. B) The related hemocompatibility image is shown in the inset. C) Whole‐blood clotting assay of mushroom extracts. D) The comparable image of the blood supernatants is shown in the inset. Values are presented as mean ± SD. Statistical significance is indicated as follows: no line (*p* > 0.05, not significant), *p ≤ 0.05, **p ≤ 0.01, ***p ≤ 0.001, and ****p ≤ 0.0001.

Many of the materials planned to be used for therapeutic purposes may enter circulation and interact with red blood cells (RBCs). A hemolysis test was performed to investigate the effect of *Lycoperdon pyriforme* on membrane damage and cell death. A low percentage of hemolytic potential (<5%) is considered critically safe for therapeutic operations, according to criteria in ASTM E2524‐08.^[^ ^38^ ^]^ The hemolytic activity test results are given in **Figure** **9**A,B. The oxidative stress effect was measured in blood samples using *Lycoperdon pyriforme* (20–1000 μg mL^−1^), and *Lycoperdon pyriforme* showed <5% hemolytic activation. It was concluded that it did not cause significant damage to RBCs.

The in vitro blood coagulation performance of mushroom extract was evaluated by the blood coagulation index (BCI). As shown in Figure 9C,D, different concentrations of mushroom extracts were incubated with blood, and the BCI index of all groups was below 10%. This showed that mushroom extract significantly increased the rate of blood coagulation. It suggests that it can be used therapeutically as a coagulant agent. To the best of our knowledge, there are no previous studies in the literature that have evaluated the hemolytic or coagulation effects of *Lycoperdon pyriforme*. Therefore, the interpretations presented here are based solely on our experimental data. While the observed hemolytic potential (<5%) suggests that the material can be considered safe according to ASTM E2524‐08 criteria, and the coagulation index values indicate a potential pro‐coagulant effect, further studies are needed to validate and expand upon these findings in comparison with standard references or related biomaterials.

### 3.2 Discussion

*Lycoperdon pyriforme* is a mushroom that is recognized and utilized by the locals of Sultan Murat Highlands for its wound‐healing properties. The present investigation, which builds on this ethnobotanical knowledge, concentrates on the extraction, purification, and characterization of *β*‐*D*‐glucan from *Lycoperdon pyriforme* and its potential role in wound healing. The results indicate that the isolated *β*‐*D*‐glucan possesses promising biological properties that could be utilized for therapeutic purposes, particularly in the promotion of wound healing.

The *β‐D*‐glucan isolated from *Lycoperdon pyriforme* in this study demonstrated non‐cytotoxic behavior, enhanced fibroblast migration, and strong hemocompatibility, aligning with prior findings reported for *β*‐glucans derived from other fungal species such as *Piptoporus betulinus*, *Schizophyllum commune*, and *Pleurotus eryngii*.^[^ ^15^, ^17^, ^30^ ^]^ In particular, de Jesus et al. (2018) showed that *β*‐glucan from *P. betulinus* promoted wound closure in vitro, which is in agreement with our scratch assay results indicating 40%–50% closure within the first 10 h at moderate concentrations.^[^ ^30^ ^]^ Similarly, Cao et al. (2021) demonstrated reduced inflammation and faster healing in patients using *Schizophyllum commune* *β*‐glucan post‐laser treatment, a finding consistent with the enhanced migration and low cytotoxicity observed in our study.^[^ ^14^ ^]^ However, unlike most of the above‐mentioned species, the *β‐D*‐glucan from *L. pyriforme* has not been previously characterized structurally with GPC, FTIR, and NMR, nor evaluated for hemolysis and clotting potential. Our study, therefore, provides a more comprehensive biochemical and biological profile of this traditionally used species. The blood coagulation enhancement observed here also resembles findings from *Pleurotus*‐based chitin‐glucan sponges,^[^ ^17^ ^]^ supporting its possible use in hemostatic applications. Compared to these species, the current results further highlight *L. pyriforme* as a regionally important but globally underexplored fungal source for wound‐healing biomaterials.

Through gel permeation chromatography (GPC), it was observed that there were two distinct peaks with average molecular weights (Mn) of 3 kDa and 104.55 kDa, indicating that the polysaccharide has a polydisperse nature. This molecular weight distribution is in accordance with the literature.^[^ ^22^ ^]^ FTIR spectroscopy confirmed the presence of distinct *β*‐glucan functional groups. Sugar and anomeric regions were clear with the signals between 1200–950 cm^−1^ and 950–750 cm^−1^. The presence of the FTIR absorption peaks at 1377.4, 1319.5, 1026.8 cm^−1^, and also the quite weak peak around 900 cm^−1^ clearly shows the characteristics of *β*‐glucan in the structure.^[^ ^25^ ^]^ In the FTIR spectrum, the ‐OH functional group signal and C‐H aliphatic signals were clear and supportive of the polysaccharide structure. In the FTIR spectra, absoption bands corresponding to amide I and II (1622 and 1556 cm^−1^) are possible signs of the presence of protein impurities. These signals can be removed by further purification. The extract in alcohol can be treated with enzymes like protease and α‐amylase to hydrolyze protein residue; however, in this process depolymerization might be observed on the structure. Also, after the process of extraction, precipitation with trichloroacetic acid, and treatment with the enzyme protease, using Sevag method can be applied to remove the possible protein residue.^[^ ^39^ ^]^

^1^H NMR analysis detected the characteristic signals of *β*‐(1 → 3) and *β*‐(1 → 6) linkages. Anomeric proton signals of glucan have appeared in the range of 4–5 ppm, which are highly influenced by the chemical moieties present in their structure.^[^ ^40^ ^]^ As evidence for the presence of the aliphatic structure of glucose *β*‐glucan, our ^1^H NMR spectrum clearly shows characteristic peaks inaccordance with the literature.^[^ ^27^ ^]^ The region between 2 and 5 ppm also shows characteristic peaks for aliphatic ‐OH protons. Anomeric signals between 3.3 and 3.8 ppm correspond to R‐*O*‐CH_3_ protons. The presence of *β*‐(1 → 3) and *β*‐(1 → 6)‐*D*‐glucan was supported by the peak at 4.59 and 4.32 ppm.^[^ ^28^ ^]^ Typical signals of *β*‐(1 → 3)‐linkages were observed in the ^1^H NMR spectrum as 4.70, 4.59, and 4.19 and 4.01 ppm. These values are also quite close to those reported in the literature.^[^ ^23^, ^30^ ^]^ One limitation of the current study is the absence of ^13^C NMR data, which could have provided additional structural confirmation of the *β‐D*‐glucan backbone, particularly with regard to the carbon framework and branching patterns. However, the combination of ^1^H NMR, FTIR, and GPC analyses allowed for sufficient preliminary structural identification. Future studies employing ^13^C NMR and 2D NMR techniques (e.g., HSQC, HMBC) would offer a more comprehensive elucidation of the *β*‐glucan structure and enhance the understanding of its structure–activity relationship.

Based on the cytotoxicity assay results, it was found that the *β*‐*D*‐glucan extract did not show any toxicity toward NIH 3T3 cells, even at high concentrations (up to 1000 μg mL^−1^). Based on the findings, it appears that the extract can be safely used in biological systems, indicating its potential for use in wound healing. Through the in vitro scratch assay, it was observed that *β*‐*D*‐glucan has a significant impact on cell migration and wound closure, indicating its potential in promoting these processes. It is worth mentioning that the groups treated with lower concentrations of *β*‐*D*‐glucan (20 μg mL^−1^, 100 μg mL^−1^, 500 μg mL^−1^) showed a faster wound closure rate compared to the control group. This suggests that *β*‐*D*‐glucan is effective in promoting cell proliferation and migration. Despite the fact that the highest concentration (1000 μg mL^−1^) exhibited a delayed initial wound closure, it ultimately achieved complete closure within 48 h, highlighting the dose‐dependent effects of *β*‐*D*‐glucan on wound healing.

The hemolytic activity assay demonstrated that *β*‐*D*‐glucan had minimal hemolytic effects (<5%) on red blood cells (RBCs), which is consistent with the ASTM E2524‐08 standard for hemocompatibility. Consequently, it is safe for therapeutic use. Additionally, the in vitro blood coagulation assay demonstrated that the *β*‐*D*‐glucan extract demonstrated a substantial increase in the rate of blood coagulation, with all groups demonstrating a blood coagulation index (BCI) below 10%. This implies that *β*‐*D*‐glucan has the potential to be a highly effective coagulant agent, which would increase its usefulness in wound care.

Although the molecular mechanisms of the effects observed in this study have not been experimentally investigated, there are studies in the literature showing that *β*‐glucans exert their biological effects by interacting with cell surface receptors such as Dectin‐1, complement receptor 3 (CR3), and Toll‐like receptors (TLR). These interactions can activate intracellular signaling pathways such as PI3K/Akt, MAPK, and NF‐κB, and these pathways regulate processes such as cell migration, immune response, and blood coagulation. *β*‐glucan can stimulate the PI3K/Akt pathway through several receptors, and the activation of this system may be pivotal in the maturation of dendritic cells produced by *β*‐glucan.^[^ ^41^ ^]^

The Dectin‐1 receptor is the most important and is highly expressed in many immunocompetent cells, such as monocytes, neutrophils, eosinophils, cutaneous cells, dendritic cells (DC), numerous T lymphocytes, and macrophages. Once it binds to the Dectin‐1 receptor, *β*‐glucan either activates other immune and non‐immune response pathways or boosts the production of many cytokines.^[^ ^15^ ^]^ Dectin‐1 is a type‐II transmembrane C‐type lectin receptor that specifically recognizes β(1 → 3)‐ and β(1 → 6)‐linked glucans present in fungal cell walls. Upon ligand binding, its cytoplasmic hemi‐ITAM motif is phosphorylated by Src family tyrosine kinases, creating a docking site for spleen tyrosine kinase (Syk). Activation of Syk initiates intracellular signaling cascades involving mitogen‐activated protein kinases (MAPKs)—such as p38, JNK, and ERK—as well as nuclear factor κB (NF‐κB). These pathways promote the production of pro‐inflammatory cytokines, including TNF‐α and IL‐6, thereby contributing to the regulation of innate immune responses such as immune cell activation, migration, and tissue repair.^[^ ^42^, ^43^ ^]^ Recent biophysical studies have demonstrated that *β*‐glucan structural features such as molecular weight and branching critically influence Dectin‐1 multimerization and activation of downstream Src–Syk signaling, leading to NF–κB and MAPK pathway engagement.^[^ ^44^ ^]^

In our study, *β‐*glucan increased fibroblast migration and coagulation even in the absence of serum, suggesting that it may have a direct effect via extracellular signaling. To ensure the most accurate evaluation, further molecular‐level analyses should be conducted based on specific molecule–receptor pairs previously identified in the literature. In line with these findings, elucidation of the specific signaling pathways underlying these effects in future studies will contribute to a better understanding of the therapeutic potential of *β*‐glucan in the field of wound healing and hemostasis.

In conclusion, the study effectively isolated and characterized *β*‐*D*‐glucan from *Lycoperdon pyriforme*, thereby demonstrating its non‐toxic, hemocompatible, and wound‐healing properties. These results emphasize the therapeutic potential of *β*‐*D*‐glucan in facilitating wound healing and establish a scientific foundation for its application in alternative medicine. Future research should concentrate on the further elucidation of the molecular mechanisms that underlie *β*‐*D*‐glucan's biological activities and the investigation of its efficacy in clinical settings.

## Conflict of Interest

The authors declare no conflict of interest.

## Author Contributions

**Elif Yavuz‐Dokgöz**: conceptualization; methodology; investigation; writing—original draft; data curation; visualization; supervision; project administration. **Meltem Güleç**: investigation;methodology; writing—original draft; data curation; visualization. **Burçin İzbudak**: investigation; formal analysis; writing original draft; visuation. **Onur Şahin**: investigation; formal analysis; methodology; writing—original draft; visualization; data curation. **Ayça Bal‐Öztürk**: investigation; methodology; writing—original draft; data curation; visualization supervision. **All authors**: writing—review and editing; validation; approval of the final manuscript.

## Supporting information

Supplementary Material

## Data Availability

The data that support the findings of this study are available on request from the corresponding author. The data are not publicly available due to privacy or ethical restrictions.
