# Supplementary figures and images for: Isolation, Characterization, and Wound‐Healing Potential of β‐D‐Glucan from Lycoperdon pyriforme Schaeff
Source: ChemistryOpen. 2025 Jul 27;14(11):e202500131. doi: 10.1002/open.202500131 (PMC12598792; doi:10.1002/open.202500131)

## MUSHROOM PICKING AREA GEOGRAPHICAL LOCATION

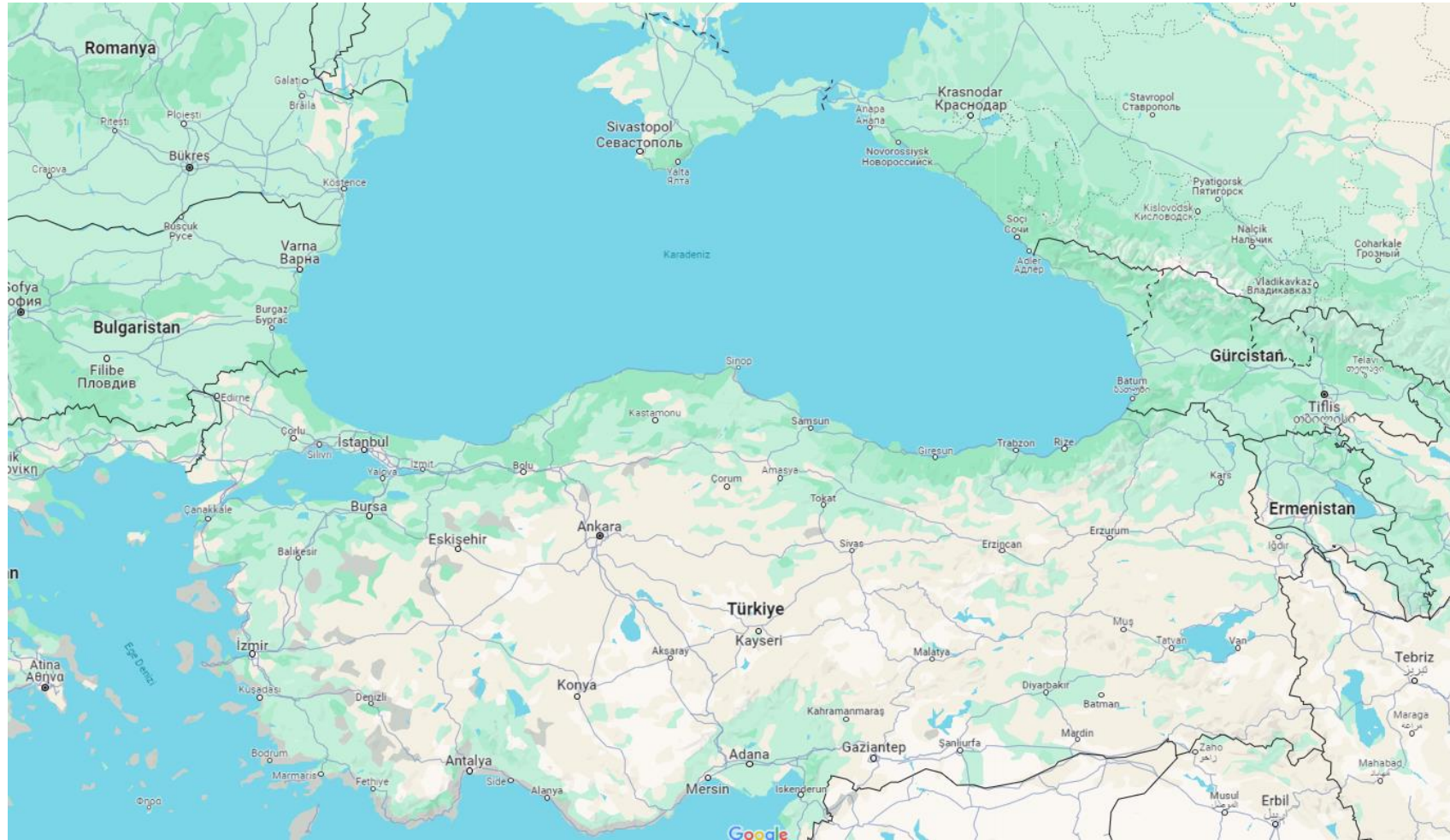

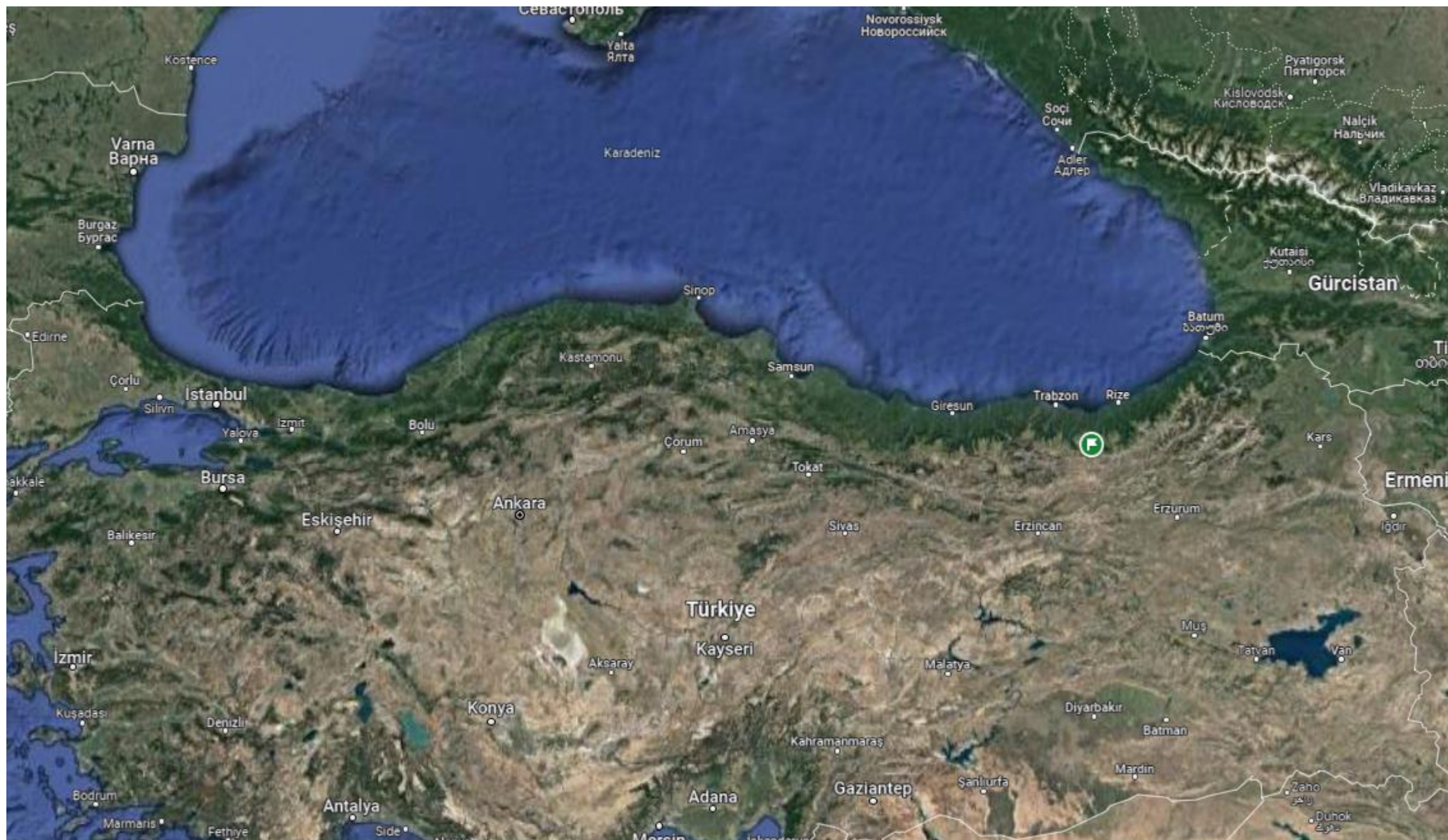

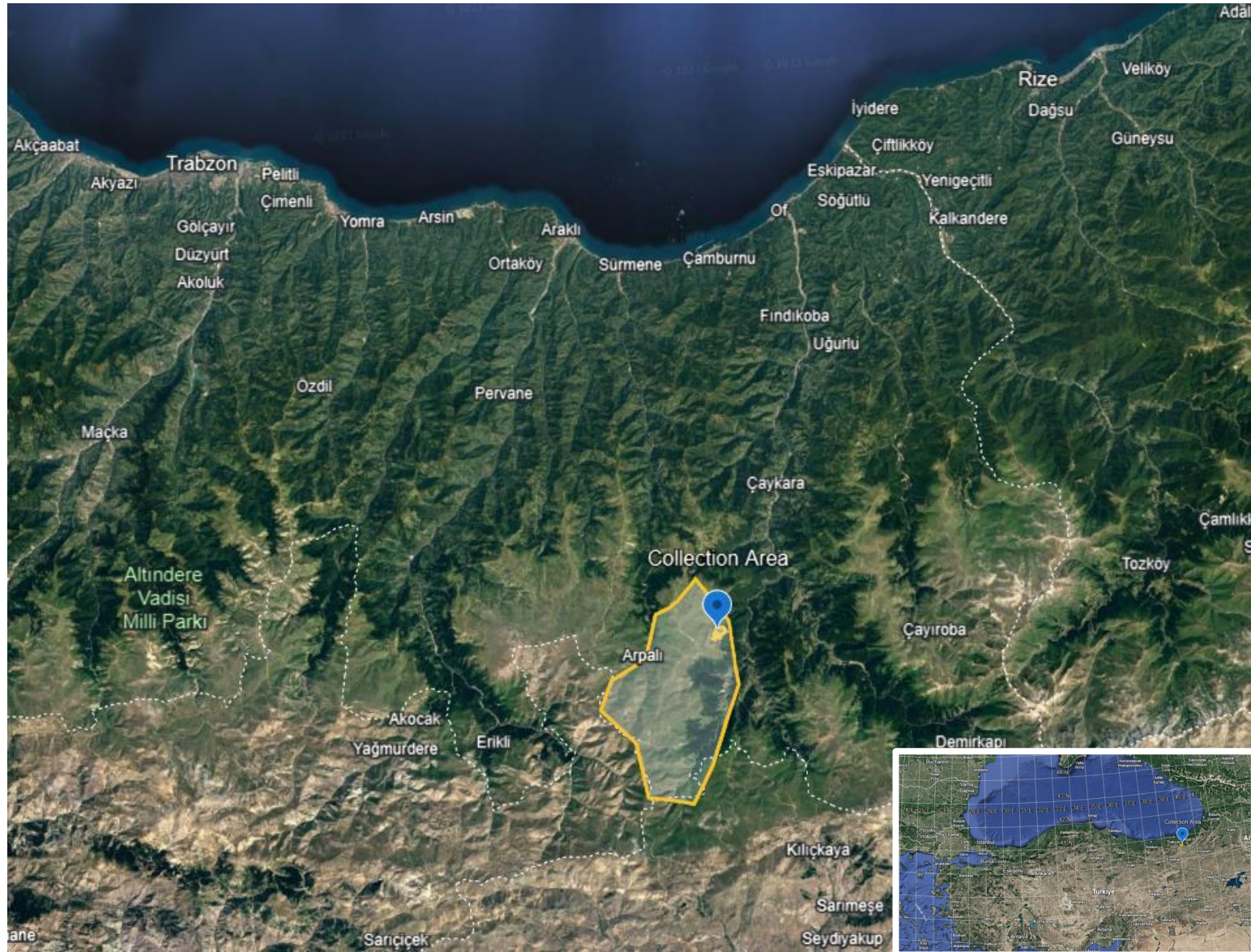

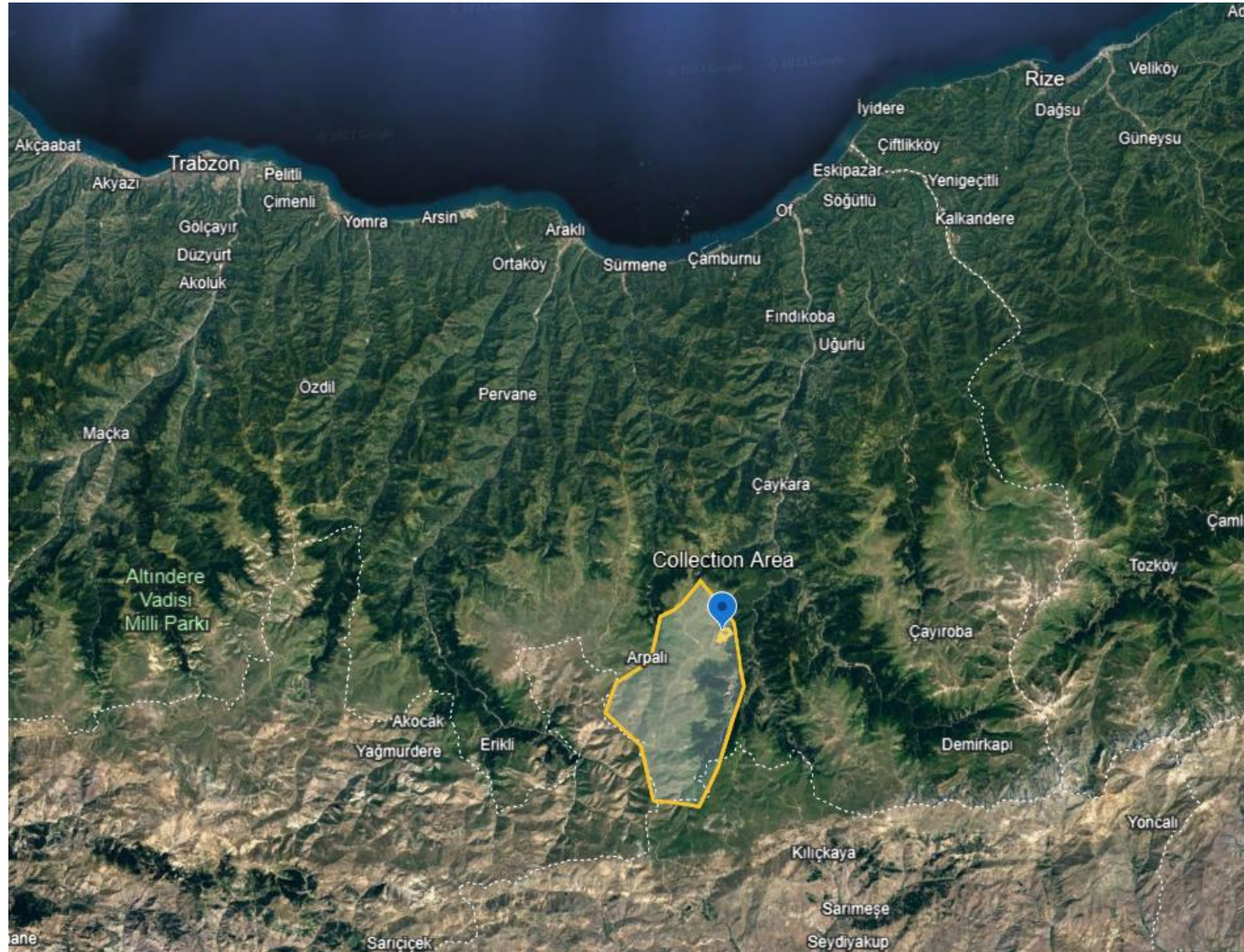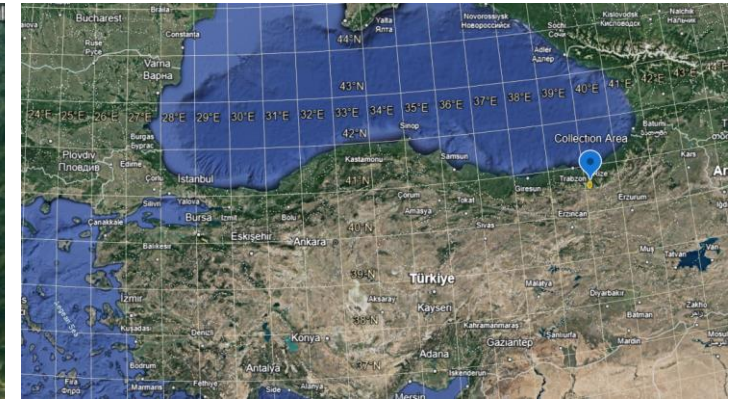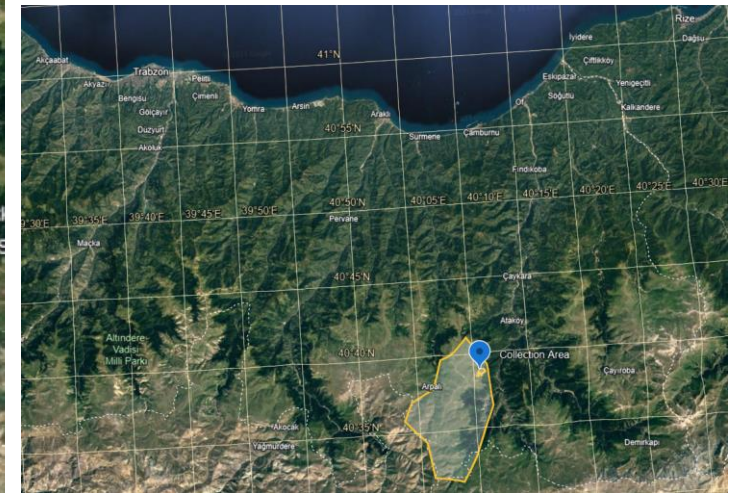

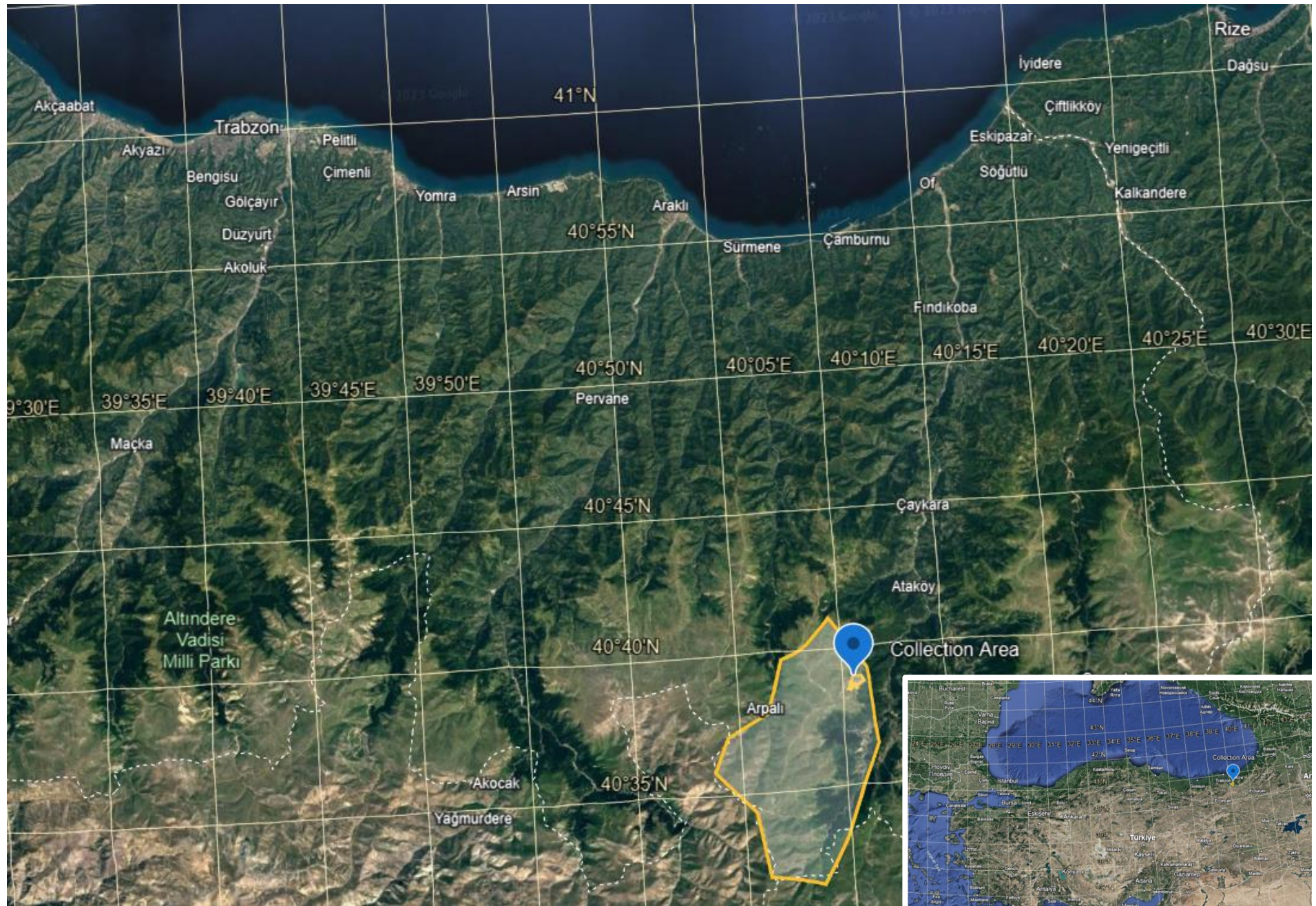

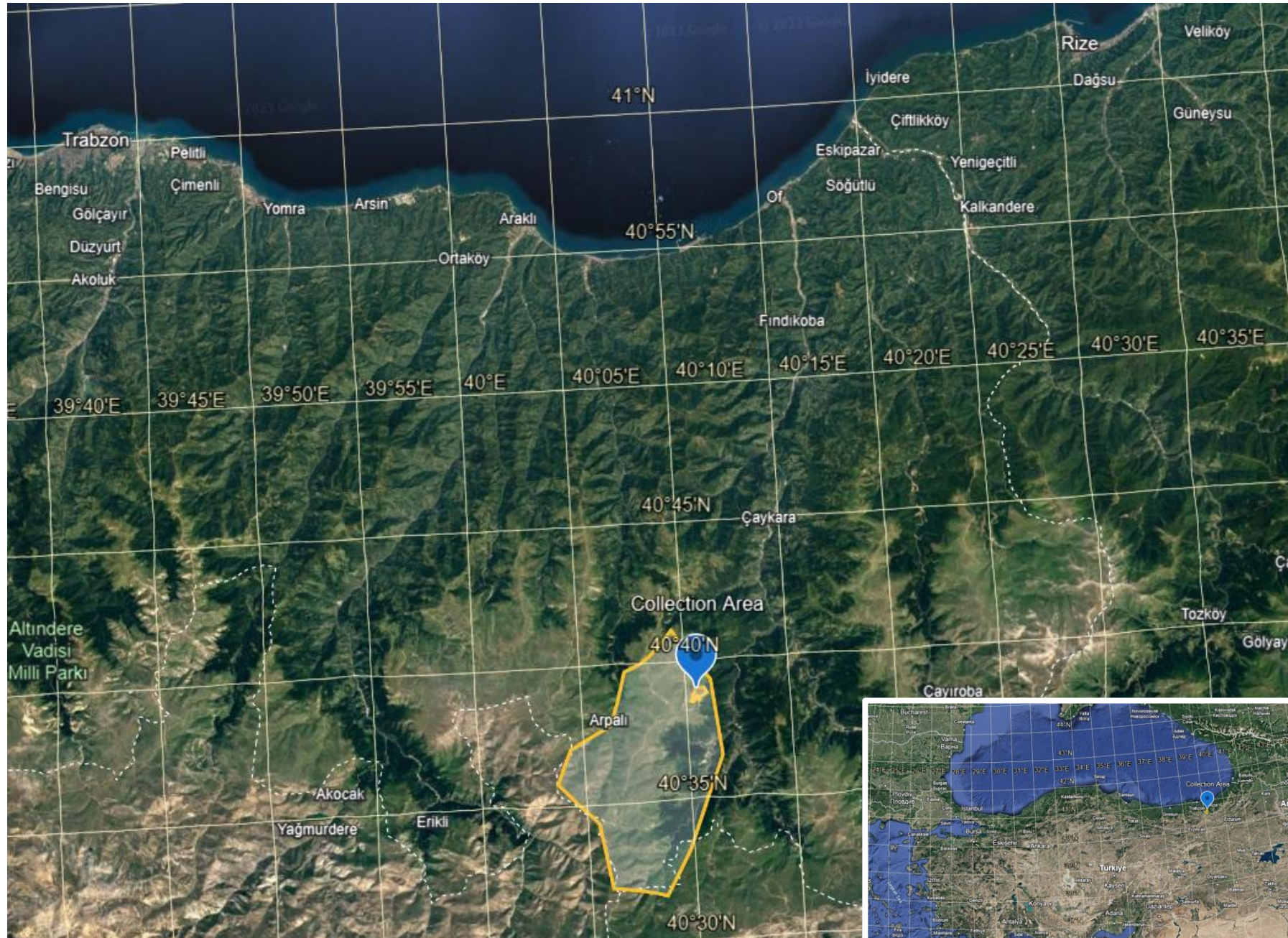

Supplement: Supplementary file 1 — Supplementary Material [file OPEN-14-e202500131-s001.pdf]
